# Accuracy of fetal fibronectin for the prediction of preterm birth in symptomatic twin pregnancies: a pilot study

**DOI:** 10.1038/s41598-018-20447-5

**Published:** 2018-02-01

**Authors:** Florent Fuchs, Clémentine Lefevre, Marie-Victoire Senat, Hervé Fernandez

**Affiliations:** 1 Department of Obstetrics and Gynecology. Hôpital Bicêtre, Assistance Publique Hôpitaux de Paris (APHP), Le Kremlin-Bicêtre, France; 2 Inserm, CESP Centre for research in Epidemiology and Population Health, U1018, Reproduction and child development, Villejuif, France; 3 Department of Obstetrics and Gynecology. CHU Montpellier, 371 Avenue du Doyen Gaston Giraud, Montpellier, France; 4 Faculté de Médecine Paris-Sud, Université Paris-Sud, 94276 Le Kremlin-Bicêtre, France

## Abstract

Our goal was to evaluate the performance of fetal fibronectin (fFN) test alone or combined with cervical length (CL), to predict spontaneous preterm birth (PTB) in symptomatic twin pregnancies. We carry out a short pilot study including all uncomplicated diamniotic twin pregnancies with symptoms of preterm labor (PTL) and intact membranes at 24–33 weeks + 6 days of gestation. Studied outcome were spontaneous delivery within 7 and 14 days of testing and spontaneous PTB at <34 and <37 weeks of gestation. Among 40 women, fFN test was positive in 3 of them (7.5%). Regardless of the outcome studied CL did not significantly predict PTB. Performance of fFN was sensitivity (66.7%), specificity (97.2%), positive predictive value (66.7%), negative predictive value (97.2%), positive likelihood ratio (LR) (24.0), and negative LR (0.3) to predict spontaneous PTB within 7 days (p = 0.01). Thus, 66.1% of patients with a positive fFN test would deliver within 7 days versus 2.4% if negative testing; starting with a pre-test probability of 7.5%. Combining CL and fFN did not enable to increase enough positive LR or decrease significantly negative LR. In conclusion, fFN test alone might have a better ability to detect spontaneous delivery within 7 days among symptomatic twin pregnancies.

## Introduction

In high-income countries, incidence of preterm birth (PTB) keeps growing, and is now affecting 11% of viable births^1^. This condition represents the main cause of death among newborns and the second one, after pneumonia, during the first five years of live. In France, according to the last national perinatal study in 2016, 7.5% of all births are born preterm, with a frequency of 6,0% and 47,5%, for singletons and multiple pregnancies respectively^2^. Meanwhile, prevalence of multiple pregnancies continues to grow with 33,7 for 1000 births in the United States in 2013, mainly because of increasing maternal age at pregnancy and use of treatments against infertility in assisted reproductive treatment^3–5^.

Twin pregnancies and prematurity are closely linked, with a PTB rate in this population of 56.6% versus 9.7% for singletons, 12 times greater (Odds ratio (OR): 12.8; 95% confidence interval (CI): 12.6–12.9). In a recent meta-analysis, the preterm delivery rate for twin pregnancies before 37, 34 and 32 weeks was 41, 13 and 7%, respectively^6^. In such a high-risk population, predictive tests are therefore mandatory, both in asymptomatic and in symptomatic population in order to appropriately time steroids administration^7^.

Ultrasound cervical length measurement and fetal fibronectin (fFN) testing in maternal cervico-vaginal secretions are the most effective tools in predicting the risk of preterm delivery for asymptomatic twin pregnancies. Conde-Agudelo *et al*. showed in a meta-analysis that the risk of PTB at <32 and <34 weeks of gestation was 42.4% and 62% respectively for a cervical length <20 mm measured between 20 and 24 weeks. A cervical length <25 mm was associated with a 26% risk of PTB at <28 weeks, while it decreased to 1.4% for a cervical length >25mm^6^. In another meta-analysis, Conde–Agudelo and Romero found that fFN had only a limited accuracy in predicting PTB at <32, <34, and <37 weeks of gestation (pooled sensitivities (Se), specificities (Sp), positive (LR+) and negative (LR−) likelihood ratios ranging from 33% to 39%, 80% to 94%, 2.0 to 5.1, and 0.7 to 0.8, respectively)^8,9^. Thus, according to these results issued from heterogeneous studies, a positive fFN is associated with a 34% risk of PTB at <32 weeks and 42% risk of PTB at <34 weeks, whereas a negative fFN decreased the risk to 6% and 17%, respectively.

Only very few data (four studies pooled in a meta-analysis) exist in the literature regarding the efficacy of these two tests in a population of twin pregnancies with symptoms of labor^10–13^. While cervical length measurement appear to be unattractive in this situation^14,15^; fFN testing would be more effective in predicting delivery within 7 days (Se, Sp, LR+ and LR−: 85%, 78%, 3.9 and 0.2 respectively) rather than within 14 days or preterm birth at <34weeks^8^. Indeed, Conde-Agudelo and Romero showed that only 1.6% of patients with negative fFN will deliver during the following week versus 24.5% with a positive test^8,9^. However, these authors mentioned that further studies are still needed to conclude firmly, because their conclusions are supported only by limited data.

The objective of this pilot study was to assess the performance of fFN in cervicovaginal secretions in a limited sample of twin pregnancies with symptoms of labor in predicting spontaneous PTB and to confront it with the addition of short cervical length.

## Materials and Methods

We carried out a double blind pilot prospective cohort study in a French tertiary care center from 1^st^ December 2015 to 31^th^ May 2016. Inclusion criteria were women older than 18 years with a monochorionic or dichorionic twin pregnancy between 24 weeks and 33 weeks +6 days gestation with symptoms of preterm labor and intact membranes. All women gave informed consent before being included in the study. Preterm labor was defined by the presence of regular uterine contractions, lasting at least 30 seconds and occurring at least three times per 10 minutes, associated with significant cervical changes on digital examination or short cervix (<25 mm) during transvaginal sonographic examination. Non-inclusion criteria were monoamniotic twin pregnancy, confirmed rupture of membranes, cervical dilatation >3 cm, prolapse membranes bulging in the vagina, cervical cerclage, vaginal bleeding, placenta previa, placental abruption, severe intrauterine growth restriction, polyhydramnios, fetal malformation, preeclampsia, or medically indicated preterm delivery before 34 weeks.

Each woman was first examined with a vaginal speculum to check for closing of the cervix or opening of the cervix with membranes at external os or presence of a prolapse amniotic sac bulging in the vagina. A first swab was rotated in the posterior fornix of the vagina and sent to the laboratory for bacteriological analysis. A second swab (QuikCheck^TM^ fFN, Hologic©) was taken also in the posterior fornix of the vagina and held for 10 seconds, then dipped into a test tube with buffer and held for another 10 seconds. Following this, a test strip was insert into the buffer for 10 minutes revealing if the test was positive (two lines), suggesting a concentration of fetal fibronectin in the cervical secretions higher than 50 ng/mL, or negative (single line). The midwife in charge of the patient performed all swab tests, but it was another midwife from another department who performed the analysis of fFN and reported the result in a masked file, blinding it for both the midwife and the obstetrician in charge of the patient. The patient was also blind to the result of the test. Vaginal swab testing were carried out either on admission or within 24 hours of admission if a sexual intercourse or a digital examination had been performed in the 24 hours before the patient’s inclusion in the study. Immediately after, transvaginal sonographic measurement of cervical length was performed according to standard protocol (empty bladder, minimal pressure, measurement of the shortest length between the internal and external os, with clearest image after 3 measurements, before and after valsalva manœuvre)^16^. Finally, an evaluation of cervical changes on digital examination was also systematically performed and Bishop score was computed. The patient was then admitted to the high-risk pregnancy department, underwent blood and urinary cytobacteriological tests, received administration of tocolytics for 48 hours, corticosteroids, and prescription of bed rest according to local protocol. Demographic, maternal and fetal characteristics were recorded. Gestational age was defined according to first trimester ultrasound scan.

Outcomes studied were spontaneous delivery within 7 and 14 days of testing and spontaneous preterm birth at <32, <34 and <37 weeks of gestation. For each outcome studied (<7days, <14days, <32 weeks, <34weeks, <37 weeks) we excluded patients with preterm delivery due to induction of labor or preterm elective cesarean delivery.

First, a descriptive analysis of the population was carried out. For variables whose distribution was normal, the results are presented in mean +/− standard deviation [extreme] otherwise, the median and the 1st and 3rd quartiles are provided.

Performance of fFN and short cervix (<15 mm, <20 mm and <25 mm) in predicting the different outcomes was evaluated. Then, we carried out a comparison of the diagnostic performance (sensitivity, specificity, positive predictive value (PPV), negative predictive value (NPV), positive and negative likelihood ratio) of short cervical length (<25 mm), fFN test and Bishop score ≥5 for the same issues. A combine analysis of cervical length and fFN was also performed: This combination test was considered positive either if cervical length was <15 mm or if cervical length was 15–24.9 mm with positive fFN test. The combination test was considered negative if cervical length was 15–24.9 mm with negative fFN test or if cervical length was ≥25 mm. Likelihood ratios for a positive test result above 10 and likelihood ratios for a negative test result below 0.1 are considered to provide strong prediction. Moderate prediction can be achieved with likelihood ratios of 5–10 and 0.1–0.2, whereas those <5 and >0.2 give only minimal prediction^17^. To compute the posttest probability of spontaneous delivery within 7 days according to fFN testing, we used the following formula: Posttest probability (%) = (LR*pretest probability)/[1      − (pretest probability*(1 − LR))]; where pretest probability correspond to PTB prevalence with 7 days.

The sample size of our pilot study was derived from the results of sensitivity and specificity for fFN testing and cervical length <25 mm in the prediction of spontaneous delivery with 7 days according to Conde-Agudelo and Romero meta-analysis^8,9^. We calculated that a sample size of 32 to 50 patients would be sufficient to detect a difference in sensitivity and specificity between those two tests with a power of 80% at an alpha level of 0.05.

Statistical analysis was performed with STATA v.13 software (Stata Corporation, College Station, TX). Our manuscript follows the STARD criteria for diagnostic accuracy studies. This study received approbation of French ethics committee under the notification number, N° HAO 10012 - NI 10009(2). All methods used were performed in accordance with the relevant guidelines and regulations for managing twin pregnancies with symptoms of preterm labor.

## Results

During the 6 months of the study, 42 patients were referred to the emergency department for symptoms of preterm labor and all accepted to enter the study. Two patients were finally lost to follow up leaving 40 women entering the study.

Population characteristics, pregnancy outcome and fFN test are presented in Table 1. Spontaneous twin pregnancy accounted for 65%, whereas 35% issued from assisted reproductive treatment (2 ovulation induction and 12 *In-Vitro* Fertilization). Dichorionic diamniotic twin represented 62.5% pregnancies. Mean gestational age at inclusion was 29.6 weeks +/−2.8 (range 24.1 to 33.9 weeks) with a median bishop score of 3 [1^st^ quartile-3^rd^ quartile: 2–4]. Mean cervical length during transvaginal ultrasonographic examination was 16,1 mm +/−8.4. Distribution of short cervix (<15 mm, <20 mm, <25 mm, and <30 mm) was as followed: 45%, 75%, 90% and 95% respectively. Fetal fibronectin test was positive in 3 patients (7,5%) and all of them had a cervix <15 mm. Median length of stay in hospital was 6 days [1^st^ quartile-3^rd^ quartile: 4–12 days].

**Table 1:** Population characteristics.

Characteristics	N = 40
Maternal age, years (mean +/− SD)	29.3 +/− 5.2
**Ethnicity, n (%)**	
Europe and Nord Africa	29 (72.5)
Central and West Africa	5 (12.5)
Asian and Indian	4 (10.0)
Other	2 (5.0)
Body Mass Index*, (mean +/−SD)	22.8 +/−4.3
<18.5 kg/m² (underweight), n (%)	9 (22.5)
18.5–24.9 kg/m² (normal), n (%)	22 (55.0)
25–29.9 kg/m² (overweight), n (%)	5 (12.5)
>30 kg/m² (obese), n (%)	4 (10.0)
Nulliparous, n (%)	29 (72.5)
Gestational age at inclusion, weeks (mean +/− SD)	29.6 +/− 2.8
**Bishop score at inclusion, n (%)** ^**†**^	
Bishop <5	31 (79.55)
Bishop ≥5	8 (20.5)
Cervical length at inclusion, mm (mean +/− SD)	16.1 +/−8.4
Positive fetal Fibronectin, n (%)	3 (7.5)
Gestational age at delivery, weeks (mean +/− SD)	34.5+/−3.5
Inclusion to delivery interval, days (median (1^st^–3^rd^ quartile))	28.7 (21.4–47.3)
Spontaneous delivery <7 days, n (%)	3 (7.5)
Spontaneous delivery <14 days, n (%)	6 (15.0)

*At the beginning of pregnancy

^†^1 missing data.

Mean gestational age at delivery was 34.5 weeks +/−3.5 with a distribution of spontaneous preterm birth <32 weeks, <34 weeks and <37weeks of 25%, 32.5% and 67.5% respectively. Median days from testing to delivery were 28.7 days [21.4–47.3] with 3 patients (7.5%) and 6 patients (15%) spontaneously delivering respectively within 7 days and 14 days of testing.

Vaginal delivery occurred 27 cases (67.5%) without any cesarean section for second twin only. Women gave birth to a first male twin or a second male twin in 21 cases (52.5%) and in 21 cases (52.5%) with a mean weight of 2148 g +/−606 and 2085 g +/−624 respectively, a median APGAR at 1/5/10 minutes respectively of 10/10/10 and 8/10/10, a mean arterial pH of 7.29 +/−0.04 and 7.27 +/−0.07 respectively and a mean lactate 3.5 +/−1.4 and 4.0 +/−4.0 respectively.

Performance of fFN test and cervical length in predicting the different outcomes are described in Table 2. A positive fFN test predicted appropriately preterm delivery regardless of studied outcome (p < 0.05) except for PTB < 37weeks (p = 0.54). On the opposite, none of the cervical length cut-off chosen accurately predicted a preterm delivery (p > 0.05).

**Table 2:** Frequency (n) and performance (p value) of positive cervical fetal Fibronectin test and short cervical length at various cut off in predicting the risk of spontaneous preterm delivery (<32 weeks, <34 weeks and <37 weeks) and the risk of spontaneous delivery within 7 days and 14 days of testing, in twin pregnancies with symptoms of preterm labor.

	<7 days (n = 3)	<14 days (n = 6)	<32 weeks (n = 10)	<34 weeks (n = 13)	<37 weeks (n = 27)
Positive cervical fetal fibronectin (n = 3)	n = 2 p = 0.01	n = 3 p = 0.002	n = 3 p = 0.01	n = 3 p = 0.03	n = 3 p = 0.54
Cervical length <15 mm (n = 18)	n = 3 p = 0.08	n = 4 p = 0.38	n = 7 p = 0.14	n = 7 p = 0.44*	n = 12 p = 0.92*
Cervical length <20 mm (n = 30)	n = 3 p = 0.56	n = 5 p = 1.0	n = 8 p = 1.0	n = 10 p = 1.0	n = 21 p = 0.70
Cervical length <25 mm (n = 36)	n = 3 p = 1.0	n = 5 p = 0.49	n = 9 p = 1.0	n = 12 p = 1.0	n = 24 p = 1.0

Tests used were Fisher exact test except *Chi-2 test.

Comparison of the diagnostic performance of Bishop score ≥5, cervical length <25 mm, positive fFN test and positive combination test to predict spontaneous preterm delivery <7 days, <14 days, <32 weeks, <34 weeks and <37 weeks is summarized in Table 3. Regardless of the outcome studied a short cervical length only provided low prediction of PTB (LR+<5 and LR− > 0.2). The bishop score only reached moderate prediction ability when studying delivery <32 weeks of gestation (LR+ = 5–10). The best predictor test was fetal fibronectin that enabled to increase or decrease significantly the risk of spontaneous delivery within 7 days, with Se, Sp, PPV, NPV, LR+ and LR− reaching 66.7%, 97.2%, 66.7%, 97.2%, 24.0 and 0.3 respectively. Therefore, 66.1% of patients with a positive fFN test would deliver within 7 days versus 2.4% if negative testing; starting with a pre-test probability of 7.5%. The combination test (fFN and short cervical length), compared to short cervical length alone, did not enable to increase enough positive LR or decrease significantly negative LR.

**Table 3:** Prediction performance of different diagnostic methods for different outcomes.

	Sensitivity (%) (95%CI)	Specificity (%) (95%CI)	PPV** (%) (95%CI)	NPV° (%) (95%CI)	LR+^†^ (95%CI)	LR−^‡^ (95%CI)
**Delivery <37 weeks (frequency = 67.5%)**						
fFN	11.5 (2.5–30.2)	100 (75.3–100)	100 (29.2–100)	36.1 (20.8–53.8)	—	0.9 (0.8–1.0)
Bishop score ≥5	19.2 (6.6–39.4)	76.9 (46.2–95)	62.5 (24.5–91.5)	32.3 (16.7–51.4)	0.8 (0.2–3.0)	1.0 (0.7–1.5)
Cervical length <25 mm	88.5 (69.8–97.6)	7.7 (0.2–36)	65.7 (47.8–80.9)	25.0 (0.6–80.6)	1.0 (0.8–1.2)	1.5 (0.2–13.0)
Positive combination test*	44.4 (25.5–64.7)	53.8 (25.1–80.8)	66.7 (41–86.7)	31.8 (13.9–54.9)	1.0 (0.5–2.0)	1.0 (0.6–1.9)
**Delivery <34 weeks (frequency = 32.5%)**						
fFN	25.0 (5.5–57.2)	100 (87.2–100)	100 (29.2–100)	75.0 (57.8–87.9)	—	0.8 (0.5–1.0)
Bishop score ≥5	41.7 (15.2–72.3)	88.9 (70.8–97.6)	62.5 (24.5–91.5)	77.4 (58.9–90.4)	3.8 (1.1–13.2)	0.7 (0.4–1.1)
Cervical length <25 mm	91.7 (61.5–99.8)	11.1 (2.4–29.2)	31.4 (16.9–49.3)	75.0 (19.4–99.4)	1.0 (0.8–1.3)	0.8 (0.1–6.5)
Positive combination test*	53.8 (25.1–80.8)	59.3 (38.8–77.6)	38.9 (17.3–64.3)	72.7 (49.8–89.3)	1.3 (0.7–2.6)	0.8 (0.4–1.5)
**Delivery <32 weeks (frequency = 25%)**						
fFN	33.3 (7.5–70.1)	100 (88.4–100)	100 (29.2–100)	83.3 (67.2–93.6)	—	0.7 (0.4–1.1)
Bishop score ≥5	55.6 (21.2–86.3)	90.0 (73.5–97.9)	62.5 (24.5–91.5)	87.1 (70.2–96.4)	5.6 (1.6–18.9)	0.5 (0.2–1.0)
Cervical length <25 mm	88.9 (51.8–99.7)	10.0 (2.11–26.5)	22.9 (10.4–40.1)	75.0 (19.4–99.4)	1.0 (0.8–1.3)	1.1 (0.1–9.4)
Positive combination test*	70.0 (34.8–93.3)	63.3 (43.9–80.1)	38.9 (17.3–64.3)	86.4 (65.1–97.1)	1.9 (1.0–3.6)	0.5 (0.2–1.3)
**Delivery <7 days (frequency = 7.5%)**						
ffn	66.7 (9.4–99.2)	97.2 (85.5–99.9)	66.7 (9.4–99.2)	97.2 (85.5–99.9)	24.0 (3.0–194)	0.3 (0.1–1.7)
Bishop score ≥5	66.7 (9.4–99.2)	83.3 (67.2–93.6)	25.0 (3.2–65.1)	96.8 (83.3–99.9)	4.0 (1.4–11.8)	0.4 (0.1–2.0)
Cervical length <25 mm	100 (29.2–100)	11.1 (3.11–26.1)	8.6 (1.8–23.1)	100 (39.8–100)	1.1 (1.0–1.3)	—
Positive combination test*	100 (29.2–100)	59.5 (42.1–75.2)	16.7 (3.6–41.4)	100 (84.6–100)	2.5 (1.7–3.6)	—
**Delivery <14 days (frequency = 15%)**						
fFN	50.0 (11.8–88.2)	100 (89.4–100)	100 (29.2–100)	91.7 (77.5–98.2)		0.5 (0.2–1.1)
Bishop score ≥5	33.3 (4.3–77.7)	81.8 (64.5–93	25.0 (3.2–65.1)	87.1 (70.2–96.4)	1.8 (0.5–7)	0.8 (0.5–1.5)
Cervical length <25 mm	83.3 (35.9–99.6)	9.1 (1.9–24.3)	14.3 (4.8–30.3)	75.0 (19.4–99.4)	0.9 (0.6–1.3)	1.8 (0.2–14.8)
Positive combination test*	66.7 (22.3–95.7)	58.8 (40.7–75.4)	22.2 (6.4–47.6)	90.9 (70.8–98.9)	1.62 (0.8–3.2)	0.6 (0.2–1.8)

*Positive test if cervical length <15 mm or if cervical length = 15–24.9 mm with positive fetal fibronectin (fFN). Negative test if cervical length ≥25 mm or if cervical length = 15–24.9 mm with negative fFN. **Positive Predictive value. °Negative predictive value. ^†^Positive Likelihood ratio. ^‡^Negative Likelihood ratio.

## Discussion

Our pilot study shows that the most accurate test to predict spontaneous preterm birth in 40 twin pregnancies with symptoms of labor was fetal fibronectin. This test gave, in our small pilot study, a better prediction than cervical length for a spontaneous delivery <7days changing the individual pretest probability of 7.5% into 66.1% in case of positive result and into 2.4% in case of negative testing.

Due to our inability to prevent preterm birth worldwide, both in singletons and twin pregnancies, researches have then focused on identifying efficient markers than could predict accurately short-term preterm delivery. Such a prediction could enable to optimally time the administration of antenatal steroids; the only drug that as proven its efficiency to reduce mortality and morbidity among newborns^7^. Among those markers ultrasonographic measurement of cervical length seemed relevant^18^. Fetal fibronectin was the first bedside test available and showed promising results, especially in singleton pregnancies regarding its high negative predictive value^19,20^. On the other hand, a positive test was not so accurate as a negative one to predict a preterm birth^21^.

Recently, several teams have performed comparable work in a population of twin pregnancies, a population at high risk of preterm birth. Combs *et al*. found in a secondary analysis of a randomized trial based on 17OHPc that a positive fFN was stronger than cervical length ≤25 mm in predicting early preterm birth in asymptomatic twins^22^. Conde-Agudelo and Romero showed in their meta-analysis in 2010 that the efficacy of fibronectin was limited in predicting delivery before 32 weeks, 34 weeks and 37 weeks for asymptomatic twin pregnancies^8,9^. Regarding symptomatic twin pregnancies, the presence of fetal fibronectin in maternal vaginal secretions was predictive of delivery within 7 days with sensitivity, specificity, PPV and NPV respectively of 71%, 64%, 26%, and 93% in the study by Deplagne *et al*.^23^. On the other hand, this test was ineffective in predicting PTB within 14 days. These results were confirmed in the meta-analysis published in 2010 with an accurate prediction of delivery within 7 days (Se, Sp, LR+ and LR−: 85%, 78%, 3.9 and 0.2 respectively)^8^.

The results of our study not only agree with the literature as a whole regarding the excellent NPV of a fFN negative test to predict delivery within 7 days of testing, but seem even more encouraging to classify a twin pregnancy as a very high-risk pregnancy in case of a positive test as evidenced by the good PPV (66.7%), the high LR+ (24.0) and therefore, the increased post-test probability of 66.1%. In our study, the fFN test was also predictive of a delivery within 14 days (Se, Sp, PPV, NPV: 50%, 100%, 100% and 91.9%, respectively, p = 0.002). However, only three patients had a positive fFN test and all of them gave birth within 2 weeks of inclusion, limiting statistical analyses. Then, a study with a larger number of patients would be needed to give more precise prediction of a PTB within 14 days.

Many studies have investigated the effectiveness of a combined test to identify pregnancies at high risk of preterm delivery. This test was mainly based on the first realization of a cervical ultrasound in all patients presenting symptoms of threatened preterm delivery and then to carry out a fFN test only when cervix shortened. Thus, the test was considered positive in cases of short cervix <15 mm, or intermediate cervical length (15–30 mm) with a positive fFN test. The combined test was considered negative if the cervix was greater than 30 mm or intermediate with no fFN detected. The combined test appeared to perform well in a population of singletons^20,24–26^; except in one study, by Rozenberg *et al*.^19^ that showed no improvement compared to the two tests taken separately.

Two studies have investigated the use of a combined test in a population of symptomatic twin pregnancies^10,23^. Deplagne *et al*. showed that the combined test was moderately effective in predicting delivery within 14 days (Se, Sp, PPV and NPV of 89%, 57%, 35% and 95% respectively, p = 0.01) while avoiding 50% fibronectin tests^23^. The study by Gonzalez *et al*. did not assess the prediction of PTB within 7 and 14 days^10^. In the same study, regarding prediction of PTB at <34 weeks and <37 weeks, the predictive values of the sequential test were not significantly different from those obtained with the individual tests. Our results, using a combined method, were more encouraging in the prediction of a delivery within 7 days after the test (Se, Sp, PPV, NPV respectively of 100%, 59.5%, 16.7% and 100%), with 52.5% of fibronectin tests avoided, while remaining, however, lower than those of fFN alone.

The strengths of our study rely on a rigorous methodology limiting therefore the risks of bias. As the number of patient was low, we could have include a consecutive cohort of twin pregnancies referred to the obstetric emergency department for symptoms of preterm labor. Only few women was lost to follow up and none declined to participate, probably due to the fact that the fFN test is a simple vaginal swab test performed immediately after bacteriological testing. The all team in charge of the patients and the patients themselves were blinded to the result of fFN test, enabling to apply the same protocol regardless of the fFN status. Regarding the outcomes evaluated, all had been defined in the study protocol at the beginning of the study and in order to evaluate properly the prediction of the test, non-spontaneous deliveries were excluded.

Another strength of the study is that the observed PTB rate was high, as expected in such a population of high-risk twin pregnancies. The PTB rate was in accordance with the one published in previous studies confirming the appropriate selection of patients. Finally, all-important outcomes were assessed in our study, both delay outcomes (<7 or 14 days) and gestational age cutoffs (<32, 34 or 37 weeks), which was not always analyzed in previous publications. In such a high-risk group, studying delay outcomes seems more appropriate than gestational cutoffs because the main goal of bedside tests is to give quick results and rapid short-term prognosis, rather than long-term prediction. This is particularly true for twins because none existing test previously published, appears to have such a good prediction ability to decide whether the patient should be hospitalized or not. Maybe fFN, according to our results might be the appropriate one.

Our pilot study has however also some limitations. First, our main limitation is the very low sample size of patient due to the pilot nature of this cohort. Such a pilot study could only give fragmentary information and draw limited conclusion needing to be confirmed in larger studies. Twin pregnancy, even frequent in our tertiary perinatal center, has still a lower incidence than singletons. Therefore, it would have been appropriate to perform a larger multicentric cohort study to validate our results. Besides, as the number of positive tests was low (3 out of 40), we were not able to retrieve precisely some LR+ or LR− according to the outcome studied. This would have probably been different with a larger sample size.

Another limitation of our study is that we did not compare qualitative and quantitative fFN test. A recent study performed in singleton found that in women with threatened preterm birth, quantitative fibronectin testing alone performs equal to the combination of cervical length and qualitative fibronectin. Possibly, the combination of quantitative fibronectin testing and cervical length increases this predictive capacity^27^. This has not been studied in symptomatic twin and might be of interest in another study.

In conclusion, qualitative cervico-vaginal fetal fibronectin test, performed in our limited pilot study of twin pregnancies with symptoms of labor, seems to predict spontaneous preterm birth within 7 days better than cervical length. Combination of fFN and cervical length does not seem to increase the predictive accuracy of both tests taken separately. A positive fFN testing increases the individual PTB probability from 7.5% to 66.1%, due to its high positive likelihood ratio of 24, whereas a negative test decreases it to 2.4%. Therefore, according to our preliminary results, performing fFN testing in case of symptomatic twins might help in their management especially when the test is positive. These results need to be confirmed in a large prospective study of symptomatic twin pregnancies.

## References

[1] Blencowe H (2012). National, regional, and worldwide estimates of preterm birth rates in the year 2010 with time trends since 1990 for selected countries: a systematic analysis and implications. Lancet.

[2] Blondel B (2017). Trends in perinatal health in metropolitan France from 1995 to 2016: Results from the French National Perinatal Surveys. J Gynecol Obstet Hum Reprod.

[3] Blondel B (2002). The impact of the increasing number of multiple births on the rates of preterm birth and low birthweight: an international study. Am J Public Health.

[4] Blondel, B., Lelong, N., Kermarrec, M., Goffinet, F. & National Coordination Group of the National Perinatal, S. Trends in perinatal health in France from 1995 to 2010. Results from the French National Perinatal Surveys. *J Gynecol Obstet Biol Reprod (Paris*) 41, e1–e15, 10.1016/j.jgyn.2012.04.014 (2012). 10.1016/j.jgyn.2012.04.014 22613118

[5] European IVFMC (2016). Assisted reproductive technology in Europe, 2011: results generated from European registers by ESHRE. Hum Reprod.

[6] Conde-Agudelo A, Romero R, Hassan SS, Yeo L (2010). Transvaginal sonographic cervical length for the prediction of spontaneous preterm birth in twin pregnancies: a systematic review and metaanalysis. Am J Obstet Gynecol.

[7] Melamed N (2016). The role of antenatal corticosteroids in twin pregnancies complicated by preterm birth. Am J Obstet Gynecol.

[8] Conde-Agudelo A, Romero R (2010). Cervicovaginal fetal fibronectin for the prediction of spontaneous preterm birth in multiple pregnancies: a systematic review and meta-analysis. J Matern Fetal Neonatal Med.

[9] Conde-Agudelo A, Romero R (2014). Prediction of preterm birth in twin gestations using biophysical and biochemical tests. Am J Obstet Gynecol.

[10] Gonzalez N (2004). [Ultrasonographic measurement of cervical length in twin pregnancies with preterm labor: comparison with singleton pregnancies]. Gynecol Obstet Fertil.

[11] Peaceman AM (1997). Fetal fibronectin as a predictor of preterm birth in patients with symptoms: a multicenter trial. Am J Obstet Gynecol.

[12] Singer E (2007). Accuracy of fetal fibronectin to predict preterm birth in twin gestations with symptoms of labor. Obstet Gynecol.

[13] Terrone DA, Rinehart BK, Kraeden U, Morrison JC (1998). Fetal fibronectin in symptomatic twin gestations. Prim Care Update Ob Gyns.

[14] Liem SM (2013). Cervical length measurement for the prediction of preterm birth in symptomatic women with a twin pregnancy: a systematic review and meta-analysis. Obstet Gynecol Int.

[15] Fuchs I, Tsoi E, Henrich W, Dudenhausen JW, Nicolaides KH (2004). Sonographic measurement of cervical length in twin pregnancies in threatened preterm labor. Ultrasound Obstet Gynecol.

[16] Berghella V, Bega G, Tolosa JE, Berghella M (2003). Ultrasound assessment of the cervix. Clinical obstetrics and gynecology.

[17] Jaeschke R, Guyatt GH, Sackett DL (1994). Users’ guides to the medical literature. III. How to use an article about a diagnostic test. B. What are the results and will they help me in caring for my patients? The Evidence-Based Medicine Working Group. Jama.

[18] Gibson JL (2004). Prediction of preterm delivery in twin pregnancy: a prospective, observational study of cervical length and fetal fibronectin testing. Ultrasound Obstet Gynecol.

[19] Rozenberg P (1997). Evaluating the risk of preterm delivery: a comparison of fetal fibronectin and transvaginal ultrasonographic measurement of cervical length. Am J Obstet Gynecol.

[20] Schmitz T (2006). Selective use of fetal fibronectin detection after cervical length measurement to predict spontaneous preterm delivery in women with preterm labor. Am J Obstet Gynecol.

[21] DeFranco EA, Lewis DF, Odibo AO (2013). Improving the screening accuracy for preterm labor: is the combination of fetal fibronectin and cervical length in symptomatic patients a useful predictor of preterm birth? A systematic review. Am J Obstet Gynecol.

[22] Combs CA, Garite TJ, Maurel K, Das A (2014). & Obstetrix Collaborative Research, N. Fetal fibronectin versus cervical length as predictors of preterm birth in twin pregnancy with or without 17-hydroxyprogesterone caproate. Am J Perinatol.

[23] Deplagne C, Maurice-Tison S, Coatleven F, Vandenbossche F, Horovitz J (2013). [Predictive value of combined fibronectin and ultrasound cervical assessment in twin pregnancies]. Gynecol Obstet Fertil.

[24] Bruijn M (2016). Quantitative fetal fibronectin testing in combination with cervical length measurement in the prediction of spontaneous preterm delivery in symptomatic women. BJOG.

[25] Gomez R (2005). Cervicovaginal fibronectin improves the prediction of preterm delivery based on sonographic cervical length in patients with preterm uterine contractions and intact membranes. Am J Obstet Gynecol.

[26] Rizzo G, Capponi A, Arduini D, Lorido C, Romanini C (1996). The value of fetal fibronectin in cervical and vaginal secretions and of ultrasonographic examination of the uterine cervix in predicting premature delivery for patients with preterm labor and intact membranes. Am J Obstet Gynecol.

[27] Bruijn MM (2016). The predictive value of quantitative fibronectin testing in combination with cervical length measurement in symptomatic women. Am J Obstet Gynecol.

